# Real-time phase-contrast MRI can be used to quantify cerebrovascular reserve capacity – a comparative study to neurovascular ultrasound in healthy subjects

**DOI:** 10.1016/j.ynirp.2026.100332

**Published:** 2026-02-25

**Authors:** Christian Horstmann, Sabine Hofer, Peter Dechent, Mathias Bähr, Ilko Maier

**Affiliations:** a Department of Neurology, University Medical Center Göttingen, Germany; b Department of Cognitive Neurology, University Medical Center Göttingen, Germany

**Keywords:** Real-time phase-contrast MRI, Neurovascular ultrasound, Intracranial blood flow, Cerebrovascular reserve capacity

## Abstract

**Background:**

Cerebrovascular reserve capacity (CVRC) is reduced in patients with advanced large artery atherosclerosis (LAA) and represents a risk factor for ischemic stroke. Aim of this study was to compare CVRC testing derived by neurovascular ultrasound (nvUS) and real-time phase-contrast MRI (RT-PC MRI).

**Methods:**

In this study 25 subjects (age 33±13 years) without any LAA were first investigated with nvUS followed by RT-PC MRI performing a standardized hyperventilation-apnea-test. CVRC was determined in the medial (MCA) and posterior cerebral artery (PCA) using transtemporal nvUS and RT-PC MRI at 25 fps temporal resolution, 0.8×0.8 mm^2^ in plane resolution and a slice thickness of 6 mm during free breathing. For statistical analysis a paired *t*-test and a pearson correlation were used at α=0.05.

**Results:**

Overall, peak-systolic velocities (PSV) and end-diastolic velocities (EDV) showed lower values for the RT-PC MRI as compared to nvUS. The PSV-decrease during hyperventilation relative to baseline was higher in RT-PC MRI measurements (22% (MCA) and 26% (PCA) for nvUS vs. 34% (MCA) and 39% (PCA) for RT-PC MRI, p<0.001), while the relative PSV-increase from hyperventilation to apnea was comparable between both modalities with a mean difference of <10% (62% (MCA) and 78% (PCA) for nvUS vs. 70% (MCA) and 84% (PCA) for RT-PC MRI, p=0.171 for MCA and p=0.324 for PCA).

**Conclusions:**

Both nvUS and RT-PC MRI based CVRC measurements showed reliable de- and increases in PSV during the standardized breathing protocol. RT-PC MRI represents a promising technique to determine CVRC in various intracranial vessels.

## Highlights

• Real-time phase-contrast MRI (RT-PC MRI) can be used to examine cerebrovascular-reserve-capacity (CVRC) during a standardized breathing protocol in various intracranial arteries.
• Compared to neurovascular ultrasound (nvUS) relative decrease of peak-systolic velocity (PSV) during hyperventilation was higher for RT-PC MRI derived measurements.
• Relative increase of PSV during apnea was comparable between RT-PC MRI and nvUS with a mean difference of <10%.
• Dynamic changes of intracranial blood flow are well detectable using RT-PC MRI.
• Quantitative blood flow velocities derived by RT-PC MRI are lower compared to nvUS derived blood flow velocities because of partial volume effects and a lower temporal resolution of RT-PC MRI.

## Graphical abstract

**Figure:**
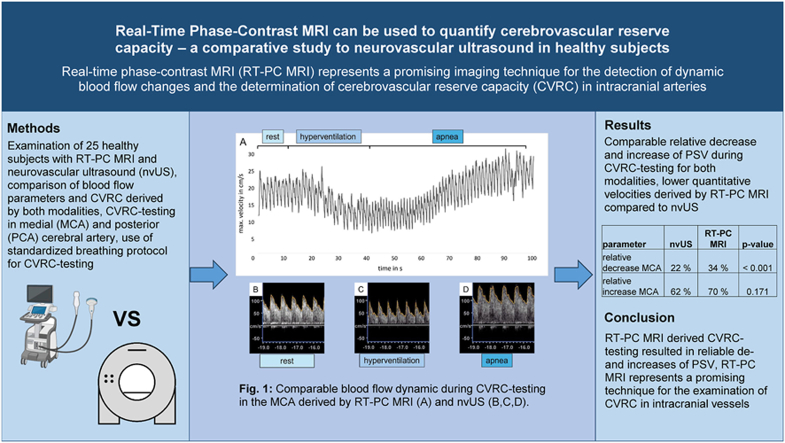

## 1 Introduction

Ischemic stroke is one of the leading causes of death and disability worldwide with an increase in incidence and consecutive socioeconomic burden (Katan and Luft, 2018; Strilciuc et al., 2021). The incidence of stroke is about 12 million new cases per year with estimated up to 25% of recurrent strokes (Esenwa and Gutierrez, 2015; Feigin et al., 2025). The latter demonstrates an urgent demand for diagnostic improvement in stroke prevention (Heit and Wintermark, 2017).

Up to 20% of all ischemic strokes are caused by large artery atherosclerosis (LAA) with consecutive narrowing and occlusions of brain supplying arteries (Marulanda-Londoño and Chaturvedi, 2016). From these, stenosis or occlusions of the carotid artery account for the majority of cases with 7-10% of all first ischemic strokes (Ritter and Tyrrell, 2012). Carotid artery stenting (CAS) and carotid endarterectomy (CEA) hereby are effective treatments for primary- and secondary prevention in this patient group (Cho et al., 2022). For the investigation of LAA, neurovascular ultrasound (nvUS) represents a well investigated diagnostic tool for determination of hemodynamic relevance and the stenotic degree in extra- as well as intracranial and collateral vessels (Purkayastha and Sorond, 2012). It also can be used to determine the cerebrovascular reserve capacity (CVRC) as a risk factor for cerebral ischemia (Douvas et al., 2016). The latter especially is relevant in patients with clinically asymptomatic stenosis and an important finding to indicate invasive treatment of the stenosis. The nvUS-derived CVRC is determined performing continuous insonation of the medial cerebral artery (MCA) while the patient is performing a standardized hyperventilation-apnea-test (Markus and Harrison, 1992).

However, the nvUS-based CVRC-testing has major limitations like insufficient transtemporal insonation windows in up to 25% of patients, limited access to all intracranial arteries and investigator dependency (Brisson et al., 2021; Purkayastha and Sorond, 2012). Concerning accessible arteries, the nvUS only allows measurements in the proximal regions of intracranial vessels, such as the M1-segment of the MCA. Further limitations of flow measurement location consider the petrous, the cisternal and the cavernous part of the intracranial carotid artery (ICA). Due to these limitations, other imaging techniques with a high spatiotemporal resolution can be an interesting addition to nvUS.

An alternative to nvUS for representation of intracranial vessels are magnetic resonance imaging techniques, such as time-of-flight magnetic resonance angiography (TOF-MRA) or phase-contrast magnetic resonance imaging (PC-MRI) (Hartung et al., 2011). In contrast to TOF-MRA, PC-MRI enables quantitative measurements of the intracranial blood flow. Two-dimensional PC-MRI (2D-PC MRI), cine PC-MRI and four-dimensional PC-MRI (4D-PC MRI) have been well investigated for quantitative blood flow measurements in intracranial arteries (Dinter et al., 2009; Morgan et al., 2023). Especially 4D-PC MRI was developed from previously mentioned 2D-PC-MRI and contributes as a promising technique for characterization of three-dimensional intracranial blood flow over time (Morgan et al., 2021). Furthermore, previous studies investigated the comparison of nvUS and mentioned PC-MRI-techniques for quantitative measurement of intracranial blood flow parameters and proved the ability of PC-MRI-techniques to measure comparable velocities with a significant correlation to nvUS (Balédent et al., 2006; Fico et al., 2023; Khan et al., 2016; Seitz et al., 2001). Nevertheless, the PC-MRI based measurements are synchronized to the heartbeat (ECG-synchronization) and often combine flow measurements over multiple cardiac cycles, with a long measurement time (Balédent et al., 2006; Gelfand et al., 2006; Seitz et al., 2001). In this cardiac-gated application techniques, the blood flow information in arrhythmic heart cycles get lost. Also, problems in the detection of dynamic blood flow changes can become relevant when using 4D-PC MRI (Morgan et al., 2021). That could lead to wrong characterization of the whole blood flow pattern during the measurement time. There are already other PC-MRI-techniques like the echo-planar-imaging PC-MRI (EPI-PC MRI), which offers a high acquisition time without the need for cardiac gating. Intracranial flow measurements and the influence of breathing on intracranial blood flow have been successfully investigated with this technique (Liu et al., 2022). However, the EPI-PC MRI is more strongly influenced by the magnetic susceptibility which can result in a more difficult post-processing (Liu et al., 2022, 2024).

Compared to aforementioned MRI-based imaging techniques, real-time phase-contrast MRI (RT-PC MRI), used in this work, provides a MRI-technique without the need for cardiac-gating and under free breathing (Joseph et al., 2012). RT-PC MRI offers a very high spatiotemporal resolution at 25 fps, 0.8×0.8 mm^2^ in plane resolution and a slice thickness of 6 mm during free breathing (Tan et al., 2017a, 2017b). Previous works investigated RT-PC MRI for flow measurements in large arteries (Joseph et al., 2012, 2014; Kowallick et al., 2014; Untenberger et al., 2016), large veins (Joseph et al., 2016, 2020) and for measurements of the cerebrospinal fluid flow (Dreha-Kulaczewski et al., 2017; Sahoo et al., 2024). Current studies investigated cerebrospinal fluid dynamics in patients with atrial fibrillation (Hofer et al., 2025). Recently it was shown that the RT-PC MRI can be used for flow velocity measurements in the extracranial carotid artery in healthy patients and can reproduce velocity measurements done with the nvUS (Maier et al., 2018). This was also investigated in patients with carotid artery stenosis (Bochert et al., 2025).

Based on these results, the aim of the present study was 1) to compare nvUS- and RT-PC MRI derived flow velocities of the intracranial vessels and 2) to compare CVRC-testing also derived by both modalities.

## 2 Material and methods

### 2.1 Subjects

In this prospective, clinical study healthy subjects without any known vascular diseases received nvUS and RT-PC MRI. Exclusion criteria were an age <18 years, pregnancy, inability to give informed consent and general exclusion criteria for MRI-measurements. Each subject has given written informed consent for study participation. This study was approved by the ethics committee of the University Medical Center Göttingen (20/7/23) and was done in accordance with the declaration of Helsinki.

### 2.2 Measurement locations

**Fig. 1:**
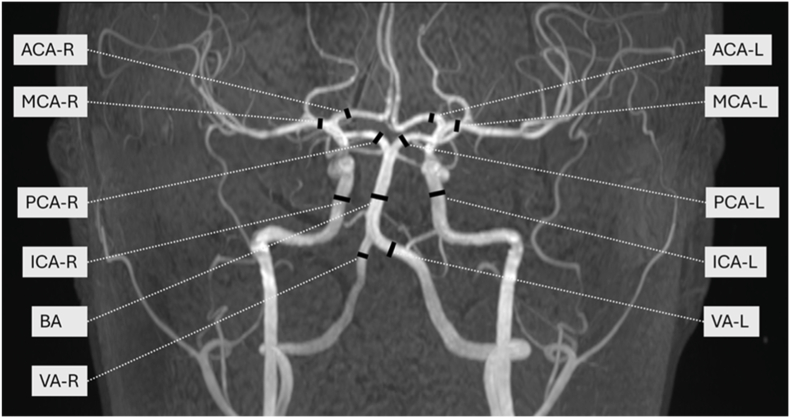
TOF-MRA of the intracranial arteries with marked locations for intracranial flow measurements with the RT-PC MRI. Measurement locations are marked as left (L) or right (R) site. MCA: medial cerebral artery; ACA: anterior cerebral artery; PCA: posterior cerebral artery; BA: basilar artery; VA: vertebral artery; ICA: intracranial carotid artery.

Each subject was first investigated with the nvUS and after that within a 2-h timeframe with RT-PC MRI. Five subjects were measured twice at two different timepoints with the same procedures to investigate reproducibility of the results. For the examination of the intracranial blood flow in this study, 11 intracranial arteries were measured (Fig. 1). Targeted arteries were the intracranial brain supplying arteries: intracranial internal carotid- (ICA), medial- (MCA), anterior- (ACA), posterior- (PCA), vertebral- (VA) and basilar artery (BA). Paired intracranial arteries like the MCA were measured on both sites. All flow measurements of the MCA, the PCA and the ACA were performed in the proximal parts of the examined arteries. The VA were measured in the distal V4-segment shortly before the fusion to the basilar artery on both sites. For RT-PC MRI measurements the ICA was examined in the intracranial, cisternal part (C1/2) shortly before the division in the MCA and the ACA. For measurements with nvUS the closest accessible intracranial part of the ICA was measured. Examined blood flow parameters were the peak-systolic velocity (PSV), the end-diastolic velocity (EDV) and the resistance index (RI). The RI enables an assessment of resistance in arterial vessels and is calculated based on measured PSV- and EDV-values (Equation (1)) (Evans et al., 1988). In addition, flow volume of the superior sagittal and the transversal venous sinus were investigated and compared to arterial inflow volume through the carotid arteries and vertebral arteries.

(1) $$RI=\frac{\left(PSV-EDV\right)}{PSV}$$

### 2.3 Neurovascular ultrasound (nvUS) of the intracranial arteries

Intracranial nvUS was performed by an investigator with more than ten years of experience who is certified by the Deutsche Gesellschaft für Ultraschall in der Medizin (DEGUM e.V.). For the examinations a Canon Aplio i700, using brightness-mode imaging, ankle corrected pulsed duplex scanning, and color-coded doppler flow imaging at 1,8-4,2 MHz, was used. Intracranial arteries were depicted in transtemporal and occipital insonation windows using color-coded duplex sonography (TCCD) mode. The nvUS system automatically identified PSV (maximum velocity of the doppler curve during the systole) and EDV (maximum of the doppler curve at the end of the diastole) in representative cardiac cycles and calculated the mean PSV from cardiac cycles in the depicted timeline. The nvUS protocol was similar to the RT-PC MRI protocol with bilaterally flow measurements of each artery and CVRC-testing in MCA and PCA at the end of the measurements including a short break of 5 min between both CVRC-testing. All subjects were in semi-supine position during the nvUS and in a supine position during the MRI scan. The duration of the nvUS protocol was variable but did not last longer than 20 min. As only healthy subjects were included in the study, no aliasing in or along the brain supplying vessels during the nvUS was detected.

### 2.4 Cerebrovascular reserve capacity

One of the main parts of this study was to investigate the dynamic changes of intracranial blood flow using the fast RT-PC MRI technique. For that, a CVRC-testing was performed in the MCA and the PCA in each subject with nvUS and RT-PC MRI. CVRC represents the ability of cerebral blood vessels to increase cerebral blood flow due to physiological stimuli e.g. increased carbon dioxide levels. CVRC also represents a marker of the ability to overcome reduced perfusion due to arterial stenosis and the risk of ischemic events (Douvas et al., 2016; Liu et al., 2019).

The breathing protocol in our study included a 10 s phase of normal breathing at the beginning with a following phase of hyperventilation for 30 s. After that, the subject holds its breath for a minimum of 30 s. To quantify the results of the CVRC-test, the breath-holding index (BHI) according to Markus and Harrison was calculated (Equation (2)) (Markus and Harrison, 1992). The BHI provides information about the relative increase of PSV and blood flow compared to the level of blood flow during normal breathing per seconds of apnea (Markus and Harrison, 1992).

(2) $$BHI=\left(\frac{\left(\frac{{PSV}_{apnea}-{PSV}_{normal\;breathing}}{{PSV}_{normal\;breathing}}\right)}{t_{apnea}}\right)\times 100$$

**Fig. 2:**
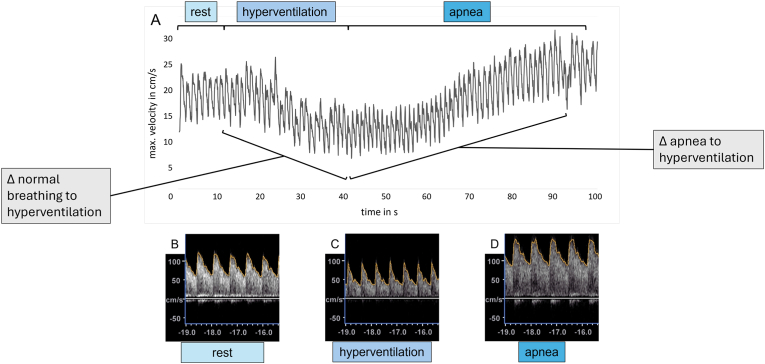
Graphic description of the cerebrovascular-reserve-capacity-testing in the MCA with the RT-PC MRI (A) and the nvUS (B, C, D). A: The y-axis represents the max. peak velocity (PSV) in cm/s and the x-axis represents the time in seconds. Compared with nvUS during normal breathing (B), hyperventilation (C) and apnea (D) both techniques show physiological arterial blood flow profiles with comparable dynamic of the PSV. Furthermore, both techniques represent a reliable decrease of the PSV during hyperventilation (second 10 to 40) and increase of the PSV during apnea (second 40 to end). Also, parameters of the CVRC-testing for the statistical evaluation are depicted. For statistical evaluation of the CVRC-measurements absolute and relative differences of PSV during the different breathing phases (rest, hyperventilation and apnea) were included. Furthermore, the BHI was calculated.

Further quantitative data from the CVRC-testing, used for statistical analysis, were the quantitative differences of PSV during the different breathing phases. Also, the relative decrease of PSV from normal breathing to hyperventilation and the relative increase of PSV from hyperventilation to end of apnea were included (Fig. 2).

### 2.5 RT-PC flow MRI

RT-PC MRI was carried out at 3T (Prisma Fit; Siemens Healthineers, Erlangen, Germany) and with a 64-channel head coil. The RT-PC MRI-technique, described previously, is based on a highly undersampled radial fast-low-angle-shot sequence (FLASH) with asymmetric gradient echoes and sequential flow encoding, using a model-based reconstruction technique (Joseph et al., 2012; Tan et al., 2017a, 2017b; Untenberger et al., 2016). Offline model-based reconstruction was used on a single graphics processing unit (GeForce GTX 580; NVIDIA, Santa Clara, CA) after real time data acquisition. For through-plane flow measurements a temporal resolution of 40 ms (25 fps) was achieved, with an in-plane resolution of 0.8×0.8 mm^2^ and a slice thickness of 6 mm. Quantitative velocity maps were calculated by a model-based reconstruction technique offering high spatiotemporal resolution (Bernstein et al., 1998; Kollmeier et al., 2022; Tan et al., 2017b). To compensate for concomitant magnetic field gradients an intrinsic Maxwell-correction was applied. There was no use of any explicit background phase correction in this study.

During MRI-investigations all subjects were in a supine position and in a semi-supine position during nvUS examination. MRI and nvUS have been started after a resting of the subject for at least 5 min in this position. The MRI-protocol started with a conventional TOF-MRA to depict the intracranial arteries. After that, flow measurements were carried out in the previously described artery-locations with a velocity-encoding-value (VENC) of 100 cm/s. The superior sagittal sinus was also measured with a VENC of 100 cm/s, transversal venous sinus were measured with a VENC of 40 cm/s. For RT-PC MRI we used predefined VENCs, which also resulted in the lack of phase wraps in the intracranial vessels and veins. Flow data were measured during free breathing and without cardiac gating. CVRC-testing was done at the end of the protocol with a short break of 5 min between the testing of the MCA and the PCA. The breathing instructions were presented to the subjects on a screen and were monitored with an abdominal belt to detect breathing movements. The duration of the MRI-protocol was 28 min.

For quantitative analysis CaFuR software (Fraunhofer Mevis, Bremen, Germany), especially developed for the analysis of RT-PC MRI flow data, was used in this work and previous studies (Bochert et al., 2025; Chitiboi et al., 2014; Maier et al., 2018; Sahoo et al., 2024). By using CaFuR a fully automated vessel segmentation and flow analysis throughout the entire time series is performed after an initial manually definition of a region of interest (ROI). The ROI is adjusted automatically through all dynamic frames and therefore also follows movements of the vessel. The automatically propagation of the ROI guarantees an accurate assessment of the vessel lumen which therefore minimizes partial volume effects (Chitiboi et al., 2014; Tan et al., 2017a, 2017b). Cardiac cycles are detected automatically. The investigator also has the ability to correct the detection of cardiac cycles. Quantitative phase contrast maps are not processed by CaFuR, therefore there is no smoothing across spatiotemporal resolution. Flow data include peak velocity (cm/s) as the highest velocity detected in the ROI, mean velocity (cm/s) averaged across the vessel area, minimum velocity (cm/s) and the area size of the vessel. Flow volume rates in ml/min are calculated automatically by CaFuR as the product of mean velocity in a ROI and the area of the ROI per time point. PSV were defined as the highest peak velocity measured in a cardiac cycle. For all arteries the mean and standard deviation of PSV and EDV of each cardiac cycle during the entire time series was calculated. For the analysis of CVRC-testing with RT-PC MRI the mean PSV from a group of manually selected cardiac cycles at the end of each breathing phase was calculated automatically by CaFuR to include the maximum effect of hyperventilation and apnea on the cerebral blood flow in the quantitative analysis.

### 2.6 Statistical analysis

The statistical analysis was done with SPSS 29 (Version 29.0.2.0 IBM SPSS Statistics, Armonk, NY, USA, https://www.ibm.com). For flow measurements PSV, EDV and the RI data were collected and analysed. For CVRC-testing the BHI- and PSV-differences during the different breathing-phases as well as the relative de- and increase of the PSV were analysed (Fig. 2). Collected data were described as mean value ± standard deviation. For the analysis a paired *t*-test and a pearson correlation analysis was carried out for each parameter at a significance level of α=0.05. For the evaluation of retest-reliability in five randomly chosen subjects, who were measured twice with nvUS and RT-PC MRI, the intraclass-correlation (ICC) was analysed using SPSS at a significance level of α=0.05. Here, a two-way mixed ICC-model with absolute agreement was used.

## 3 Results

Twenty-five healthy subjects (mean age 33.4±12.8 years, 12 subjects (48%) were male) were included in this study. Image quality on magnitude and phase-contrast maps was sufficient for a full evaluation of all intracranial arteries in 10 (40%) subjects, at least one intracranial vessel was not evaluable in 8 (32%) subjects, at least two in 6 (24%) and more than three in only 1 (4%) subject using RT-PC MRI (Table 1). Smaller and hypoplastic intracranial arteries were more likely to be non-evaluable due to image resolution. Larger arteries like the BA and intracranial ICA were evaluable in 100% of the cases. CVRC-testing with the RT-PC MRI was evaluable in all 25 subjects in both vessels, the MCA and the PCA. All arteries in every subject were evaluable using nvUS. The pixel size for flow measurements in intracranial arteries derived by RT-PC MRI was 0.64 mm^2^, as the spatial resolution was 0.8×0.8 mm^2^. By using CaFuR a ROI was individually defined for each vessel, as the size of the different vessels were variable for each subject. The areas of the ROI ranged from 1.92 mm^2^ in the small vessels like the PCA to 9.6 mm^2^ in larger vessels like the ICA. Therefore, the number of pixels included in different ROIs also ranged from 3 to 4 pixels for very small vessels up to 15 pixels for larger vessels.

**Table 1:** Evaluable vessels of the flow measurements with the RT-PC MRI.

vessel-location	evaluable RT-PC MRI measurements
MCA-L	22 (88%)
MCA-R	20 (80%)
ACA-L	19 (76%)
ACA-R	20 (80%)
PCA-L	24 (96%)
PCA-R	24 (96%)
BA	25 (100%)
V4-L	23 (92%)
V4-R	25 (100%)
ICA-L	25 (100%)
ICA-R	25 (100%)
subjects with all vessels evaluable	10 (40%)
subjects with one non-evaluable vessel	8 (32%)
subjects with two non-evaluable vessels	6 (24%)
subjects with three non-evaluable vessels	1 (4%)

Vessel locations are given as left (L) or right site (R). RT-PC MRI: real-time phase-contrast magnetic resonance imaging; MCA: medial cerebral artery; ACA: anterior cerebral artery; PCA: posterior cerebral artery; BA: basilar artery; VA: vertebral artery; ICA: intracranial carotid artery.

### 3.1 Intracranial flow measurements

Overall, PSV, EDV and RI, were significantly lower (p<0.001) using RT-PC MRI compared to nvUS in all investigated arteries (Table 2, Supplementary Table 1). The mean difference between both techniques for PSV, EDV and RI averaged over all arteries was 60.1±26.3 cm/s for PSV, 21.9±11.6 cm/s for EDV and 0.2±0.1 for RI. RT-PC MRI derived results were 70.1%±11,8% lower for PSV, 58.8%±14.8% lower for EDV and 29.8%±10.6% lower for RI. Measured PSV and EDV showed larger differences between both techniques especially in small intracranial vessels, like MCA, ACA or PCA, with a non-linear anatomy (RT-PC MRI measurements: 79.6% lower for MCA, 72.6% lower for ACA, 85% lower for PCA). Measured PSV and EDV showed more comparable results with smaller quantitative differences in arteries like the ICA or the BA, with a more linear structure. (RT-PC MRI measurements: 61.6% lower for ICA, 49.3% lower for BA). There was no significant correlation for PSV, EDV and RI between RT-PC MRI and nvUS (Supplementary Table 2).

**Table 2:** Paired *t*-test of PSV in the intracranial vessels with the RT-PC MRI and nvUS.

vessel-location	nvUS (mean cm/s ± standard deviation)	RT-PC MRI (mean cm/s ± standard deviation)	difference (mean cm/s ± standard deviation)	p-value	95%-confidence-interval
MCA-L	126.8±22.6	29.1±11.6	97.7±26.5	<0.001	86, 109.4
MCA-R	125.7±29.5	22.4±8.3	103.3±29.8	<0.001	89.3, 117.2
ACA-L	70.8±21	17.3±4.4	53.5±20.2	<0.001	43.8, 63.3
ACA-R	57.9±20.9	17.7±5.6	40.2±21.7	<0.001	30.1, 50.4
PCA-L	97.7±27.7	14.8±4.2	82.9±27	<0.001	71.5, 94.3
PCA-R	96.9±28.7	14.3±4.2	82.6±28.6	<0.001	70.5, 94.7
BA	68.3±20.1	34.7±10.7	33.7±22	<0.001	24.6, 42.8
VA-L	58.5±18.9	21.3±9.2	37.1±18.1	<0.001	29.3, 44.9
VA-R	58.5±20.3	23±8.8	35.5±20.5	<0.001	27.1, 44
ICA-L	77.1±21.1	29.3±11.4	47.8±19.4	<0.001	39.8, 55.8
ICA-R	75.9±16.4	29.5±10.4	46.5±19.5	<0.001	54.5, 11.9

Values are described as mean ± standard deviation. Vessel locations are given as left (L) or right site (R). RT-PC MRI: real-time phase-contrast magnetic resonance imaging; nvUS: neurovascular ultrasound; PSV: peak-systolic velocity; MCA: medial cerebral artery; ACA: anterior cerebral artery; PCA: posterior cerebral artery; BA: basilar artery; VA: vertebral artery; ICA: intracranial carotid artery.

Measurement results of the PSV were reproducible in five subjects receiving RT-PC MRI twice. The inter class correlation (ICC) revealed a good correlation for the measurement of PSV averaged overall vessels during normal breathing (Supplementary Table 3). For PSV derived by RT-PC MRI the ICC was 0.743 (p<0.001). For PSV derived by nvUS the ICC was 0.886 (p<0.001).

### 3.2 Cerebrovascular-reserve-capacity-testing

**Fig. 3:**
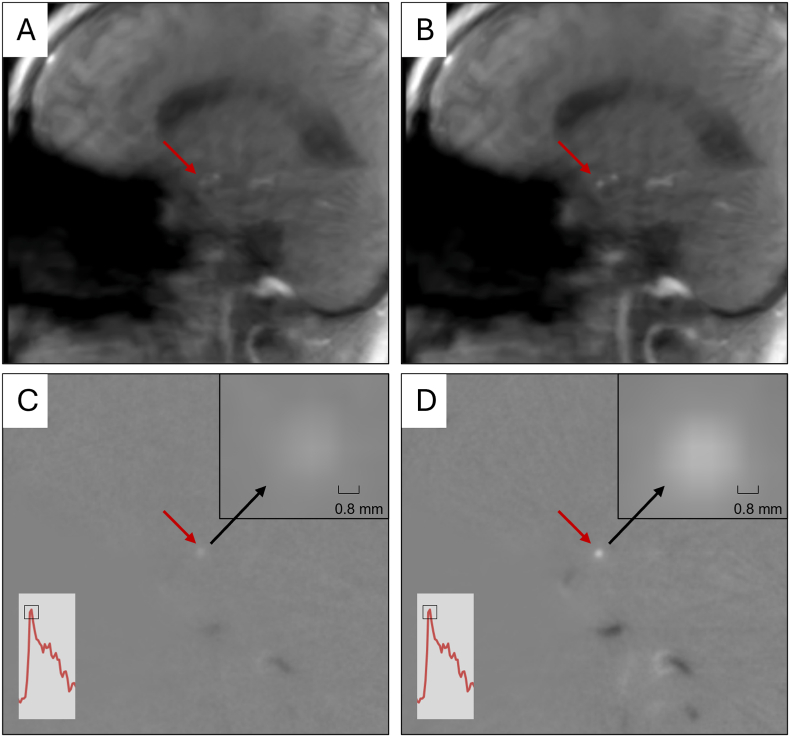
Magnitude- (A, B) and phase-contrast-images (C, D) from the RT-PC MRI during systolic phase of a single cardiac cycle at the end of hyperventilation (A, C) and at the end of apnea (B, D) in CVRC-testing of the MCA. The signal of the MCA is more hyperintense during the apnea-phase (B, D) compared to the hyperventilation-phase (A, C), because of vasodilation and therefore increased blood flow. Furthermore, the MCA during hyperventilation (C) and apnea (D) in phase-contrast images is represented in detailed zoomed-in view. The predefined area of the ROI during this measurement was 5.76 mm^2^. Both magnitude- and phase-contrast-images represent the systolic phase of one cardiac cycle at the end of hyperventilation and at the end of apnea.

CVRC-testing resulted in comparable blood flow profiles during the different breathing manoeuvres (normal breathing, hyperventilation, apnea). During the breathing protocol, a decrease of flow velocities was observed during the hyperventilation- and an increase in blood flow velocities during the apnea-phase above the level of flow velocity during normal breathing at the beginning. Measurements with both techniques resulted in an adequate decrease of blood flow and an adequate increase of blood flow with similar blood flow profiles over the whole breathing protocol (Fig. 2). Phase-contrast and magnitude maps depicted MCA and PCA with decreased intensity during hyperventilation and increased intensity during apnea (Fig. 3). Here, a video of the MCA during CVRC-testing gives interesting insights into the intensity changes due to blood flow changes during standardized breathing protocol (Video 1).

**Fig. 4:**
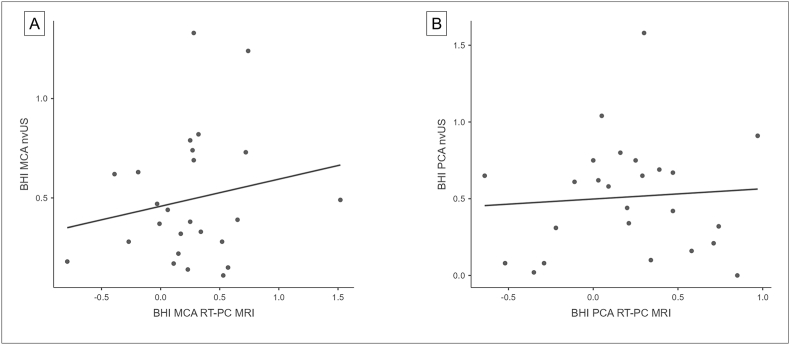
Scatter plots for BHI calculated based on CVRC-testing in RT-PC MRI and nvUS for MCA (A) and PCA (B). Calculated BHI-values resulted in quantitative differences between both techniques. The BHI-values for CVRC-testing in nvUS were quantitatively higher compared to RT-PC MRI derived BHI.

For statistical evaluation quantitative and relative differences of the blood flow between the different phases were used. The quantitative differences of PSV during different phases of the breathing protocol (from normal breathing to hyperventilation, from hyperventilation to apnea and the difference between normal breathing and apnea) were significantly higher for the nvUS measurements compared to the RT-PC MRI measurements for both vessels, the MCA and the PCA (Table 3). Also, BHI was significantly higher for measurements with the nvUS (Fig. 4). Relative differences during the breathing phases were comparable between both techniques. Relative decrease of the PSV from normal breathing to the end of hyperventilation was significantly higher for the MRI-measurements, with 34% decrease in the MCA and 39% decrease in the PCA for MRI measurements and with 22% decrease in the MCA as well as 26% decrease in the PCA during nvUS-measurements (p<0.001 for both vessels). Relative increase from the end of hyperventilation to the end of apnea was comparable between both techniques with 70% increase in the MCA and 84% increase in the PCA for MRI-measurements and 62% increase in the MCA as well as 78% increase in the PCA for nvUS measurements (p=0.171 for MCA; p=0.324 for PCA).

**Table 3:** Paired *t*-test of PSV-differences and BHI-differences in CVRC-testing with RT-PC MRI and nvUS.

parameter	nvUS (mean in cm/s ± standard deviation)	RT-PC MRI (mean in cm/s ± standard deviation)	difference (mean in cm/s ± standard deviation)	p-value	95%-confidence-interval
Δ normal breathing/hyperventilation MCA	26.5±11.4	9.1±4.9	17.5±11.4	<0.001	12.8, 22.2
Δ normal breathing/hyperventilation PCA	22.7±10.1	5.4±2	17.3±10.3	<0.001	13.1, 21.6
Δ apnea/hyperventilation MCA	55.3±16.8	11.7±6.5	43.6±18.7	<0.001	35.9, 51.3
Δ apnea/hyperventilation PCA	48.7±19.2	6.6±2.5	42.1±18.4	<0.001	34.5, 49.7
Δ apnea/normal breathing MCA	28.8±16.2	2.7±4.3	26.1±17.2	<0.001	19, 33.2
Δ apnea/normal breathing PCA	25.9±19.1	1.2±2.1	24.8±18.8	<0.001	17, 32.5
BHI MCA	0.5±0.3	0.3±0.4	0.2±0.5	0.023	0, 0.5
BHI PCA	0.5±0.4	0.2±0.4	0.3±0.5	0.007	0.1, 0.5
relative decrease PSV normal breathing to hyperventilation MCA	22%	34%	12%	<0.001	6, 17
relative decrease PSV normal breathing to hyperventilation PCA	26%	39%	13%	<0.001	6, 19
relative increase PSV hyperventilation to apnea MCA	62%	70%	9%	0.171	−10, 27
relative increase PSV hyperventilation to apnea PCA	78%	84%	6%	0.324	−22, 35

Evaluation of the CVRC-testing at a significance level of α=0.05. Values are averaged across subjects and described as mean ± standard deviation, except the relative PSV increase and decrease, which is given as a percentage value. RT-PC MRI: real-time phase-contrast magnetic resonance imaging; nvUS: neurovascular ultrasound; PSV: peak-systolic velocity; BHI: breath-holding index; MCA: medial cerebral artery; PCA: posterior cerebral artery.

### 3.3 Comparison of arterial inflow volume and venous outflow volume using RT-PC MRI

Measured total arterial inflow volumes (flow volumes of the intracranial internal carotid artery on both sites plus of the vertebral artery on both sites; 434.4±156.2 ml/min) were comparable to venous outflow volumes (flow volumes of transversal sinus on both sites: 465.6±121 ml/min, p=0.454; Supplementary Table 4). Furthermore, flow velocities and volumes of venous sinus measured with RT-PC MRI were comparable to venous flow measurements derived by other imaging techniques such as 2D-PC MRI (Mehta et al., 2000; Stoquart-Elsankari et al., 2009) (Supplementary Table 5). There was no significant correlation between arterial inflow volume and venous outflow volume measured by RT-PC MRI. Both arterial inflow and venous outflow volumes were evaluated during normal breathing.

## 4 Discussion

To date, the present study represents the first investigation of cerebral blood flow with the RT-PC MRI technique, used in this work. In our study, we could demonstrate that RT-PC MRI yielded physiological blood flow profiles, supporting its potential for detecting arterial blood flow in intracranial arteries. However, a clear discrepancy becomes evident when examining the quantitative flow velocity data obtained from 11 intracranial arteries and comparing these quantitative differences with previous studies investigating RT-PC MRI derived flow velocities in the extracranial arteries (Bochert et al., 2025; Maier et al., 2018). RT-PC MRI consistently showed lower blood flow velocities than nvUS. Our study revealed even greater quantitative differences in flow velocities for smaller and more distal intracranial arteries than earlier investigations focusing on the carotid artery. Several factors might explain this vessel-size dependent differences between both modalities:

First, intracranial arteries have a more curvy and non-linear structure in different anatomic planes compared to arteries that are more proximal to the heart like the extracranial carotid artery. Furthermore, a normal hexagonal anatomy of the circle of Willis is only present in estimated 20-31% of the people (Berghout et al., 2025; Jones et al., 2021). The use of RT-PC MRI for blood flow measurements requires a correct orthogonal measurement in a 90° angle of the vessel, which therefore is more difficult for smaller vessels with a non-linear structure like intracranial vessels. To reduce this problem, orthogonal measurements of the targeted vessel were positioned in the coronal, sagittal and transversal plane. However, for some vessels there was no perfect orthogonal measurement, which ended up in inaccurate vessel representation and partial volume effects. The slice position of the RT-PC MRI measurement was based on a TOF-MRA done at the beginning of the measurement protocol. So, advanced investigation time and because of that small movements of the subjects could also have led to incorrect positioning of the measurement location. Furthermore, in contrast to nvUS and based on different fundamental physical measurement techniques (Bochert et al., 2025), RT-PC MRI also shows requirement for strictly laminar flow to guarantee reliable measurements of quantitative flow velocities for small vessels. These conditions are not met in an in-vivo model of the brain supplying arteries, as intracranial vessels are not straight and show a lot of branches and curves. Also, subjects were investigated in supine position in the MRI scanner and in a semi-supine position during nvUS, which might have led to different flow velocities in the intracranial vessels. However, this effect is likely to be neglectable as positioning to a 15-30° head elevation only moderately reduces flow velocities due to orthostatic mechanisms. In addition, flow velocities remain within the normal physiological range in healthy subjects with intact cerebral autoregulation and are unlikely to explain the significant quantitative differences in flow velocity measurements between both techniques (Deseoe et al., 2023; Schwarz et al., 2002).

Second, partial volume effects might have more influence when measuring through-plane flow in smaller, intracranial arteries. Flow measurements of intracranial blood flow using nvUS are done placing the doppler volume longitudinally and angle corrected in the specified artery location, which reduces partial volume effects threw movement of vessel walls or surrounding tissue to a minimum (Meola et al., 2021; Purkayastha and Sorond, 2012). In contrast to that during evaluation of MRI measurements partial volume effects must be considered (Bouillot et al., 2018). When defining the vessel lumen, based on the hyperintense signal of the vessel on the phase-contrast map, the beginning of the vessel wall cannot clearly be differentiated. Improved vessel segmentation algorithms, used in this work, already reduced this problem (Chitiboi et al., 2014). However, separate voxel also including information of slower moving tissue like the vessel wall can also led to lower blood flow velocities, as previously described (Bochert et al., 2025). Furthermore, small vessels like the ACA or PCA were also represented by a lower number of pixels included in the individual ROI compared to the larger vessels like the ICA. Here, partial volume effects are exacerbated, as each pixel might include more proportion of non-blood signal, which leads to a stronger variability of flow results (Bouillot et al., 2018).

Third, RT-PC MRI flow velocity measurements have a lower temporal resolution compared to nvUS. During this study PSV-values were used to compare RT-PC MRI and nvUS for the measurement of intracranial blood flow velocities. Regarding lower quantitative PSV-values of intracranial arteries derived by RT-PC MRI, the temporal resolution of both techniques must also be considered. As already discussed in previous studies, compared to nvUS, RT-PC MRI has a lower temporal resolution with 40 ms per image, which means 25 fps (Bochert et al., 2025; Maier et al., 2018). In contrast to that nvUS represents a much higher temporal resolution, regarding the propagation speed of ultrasound waves in soft tissue about 1541 m/s and the short distance from the investigated vessel to the ultrasound probe (Purkayastha and Sorond, 2012). So, peak velocities during the systole of a cardiac cycle might be not accurately measured by RT-PC MRI. Compared to the previously investigated extracranial carotid artery (Bochert et al., 2025; Maier et al., 2018), this factor becomes even more relevant for flow measurements of small intracranial vessels, with a higher resistance and a short maximum peak velocity. This might explain larger quantitative discrepancies between the measured peak velocities derived by RT-PC MRI compared to the previous studies.

Regarding total cerebral blood flow volumes derived by RT-PC MRI, both total arterial inflow and venous outflow were comparable, but did not result in a significant correlation. This can be explained by multiple complex ways of venous outflow, that are not represented in the flow measurements of sagittal sinus and transversal sinus (De Vis et al., 2018; Stoquart-Elsankari et al., 2009). On the one hand, the venous outflow was only measured in the three major cerebral sinuses, neglecting all other outflow veins, as well as a high degree of anatomical variations and, on the other hand, there was a low number of investigated subjects in this exploratory analysis. Furthermore, total arterial blood flow was underestimated by RT-PC MRI compared to previous works investigating cerebral blood flow in healthy subjects (Zarrinkoob et al., 2015). This can be explained by the aforementioned problems that also led to lower quantitative flow velocities. However, total inflow volumes of intracranial arteries and the total venous outflow volumes derived by RT-PC MRI were comparable. Therefore, the use of RT-PC MRI may be interesting for the diagnosis of other cerebrovascular diseases like arteriovenous malformations to detect discrepancies between arterial inflow and venous outflow, as well as risk assessment of sinus vein thrombosis or stenosis. In addition, dynamic flow measurements of the venous sinus derived by RT-PC MRI could also be useful to diagnose and investigate diseases associated with in- or decreased intracranial pressure like the idiopathic intracranial hypertension or cerebrospinal fluid leak syndrome.

Concerning the measurement of CVRC previously investigated measurement of CVRC with blood oxygenation level dependent MRI (BOLD-MRI) and quantitative PC-MRI resulted in promising methods to measure CVRC (Caputi et al., 2014; Liu et al., 2021; Stickland et al., 2021). However, BOLD-MRI as an often used MRI-technique to detect CVRC requires a complex detection of different metabolic and hemodynamic blood flow parameters including e.g. cerebral blood flow, cerebral blood volume, the cerebral metabolic rate of oxygen and the haematocrit (Liu et al., 2019). In contrast, RT-PC MRI, used here, offers a direct measurement of intracranial blood flow based on a very high spatiotemporal resolution and enables non-cardiac gated measurements of dynamic changes in blood flow during normal breathing. There are no previous studies that investigated RT-PC MRI, used in this work, for the measurement of CVRC and compared the results to nvUS.

The examination of CVRC is used to investigate vasoconstriction and -dilatation under hyperventilation and apnea caused by a de- and increase of arterial carbon dioxide (Douvas et al., 2016). A reduction or lack of change of blood flow velocities is a widely accepted sign of a diminished CVRC, caused by proximal stenosis of brain supplying arteries and insufficient collateral status, again increasing the risk for ischemic stroke (Wu et al., 2018; Zavoreo and Demarin, 2010). In this study a hyperventilation-apnea-test was used to increase the blood flow during the examination. The breath-holding method is a well investigated, non-invasive method, which can be easily performed without long preparation. During this test the patient has to do a phase of apnea, in which the carbon dioxide levels are increasing and intracranial vessels react with a vasodilatation and increased blood flow (Markus and Harrison, 1992; Stoll et al., 1994).

Using both modalities RT-PC MRI and nvUS, the relative increase of PSV from hyperventilation to apnea was comparable in the MCA and PCA. Despite aforementioned partial volume effects, CVRC-testing derived by RT-PC MRI resulted in typical flow dynamics in both MCA and PCA for all 25 subjects. After the normal breathing phase at the beginning of the test, RT-PC MRI recorded a decrease of PSV during hyperventilation, due to vasoconstriction and decreasing carbon dioxide levels. Measurements in the following apnea-phase showed an increase of PSV due to vasodilatation and increasing carbon dioxide levels (Fig. 2). This can also be seen in form of a lower hyperintense signal of the MCA during hyperventilation and a significantly more hyperintense signal of the MCA during the apnea-phase (Fig. 3). In comparison to nvUS quantitative differences of PSV during the different breathing phases were lower for RT-PC MRI measurements. This can be explained by already discussed lower MRI derived PSV-values compared to nvUS derived measurements. In contrast to that, relative de- and increases of PSV during the breathing protocol were comparable between both techniques, what demonstrates comparability between both techniques for the measurement of CVRC. Regarding the BHI, nvUS measurements resulted in quantitatively higher BHI. Main reason here might be the specified measurement time of the RT-PC MRI, that in some cases lead to no significantly higher PSV at the end of apnea compared to the PSV level during normal breathing. So, in some cases the increase of PSV during apnea simply could not significantly exceed the PSV during normal breathing for MRI-measurements and the BHI represents the relative increase of PSV per seconds of apnea compared to the PSV during normal breathing (Markus and Harrison, 1992; Stoll et al., 1994). An interesting solution here might be a calculation of the relative increase of PSV per second of apnea compared to PSV at the end of hyperventilation or the use of a longer measurement time for MRI-measurements. Also, an influence of excitement at the start of CVRC-testing must be observed when the subject is lying in the MRI for a certain time and then is requested to do breathing maneuvers. Thus, increased blood pressure could lead to higher PSV levels during normal breathing at the beginning of the test. However, CVRC-testing resulted in valid measurements of hemodynamic changes in intracranial arteries derived by the RT-PC MRI.

Regarding the parameters to evaluate CVRC and calculate BHI in this study, future studies should also consider using mean flow metrics rather than PSV-values. Due to previously investigated PSV measured with both techniques for extracranial vessels, PSV was chosen as the preferred parameter in this study (Maier et al., 2018). Flow measurements during normal breathing in this study resulted in lower quantitative PSV compared to nvUS, with larger quantitative differences compared to previous studies, illustrating now the PSV as a potentially imbalanced parameter for RT-PC MRI, especially for intracranial flow measurements (Bochert et al., 2025; Maier et al., 2018). The main reasons are partial volume effects and a lower temporal resolution to nvUS, that lead to an underestimation of PSV. Different other studies suggest using mean flow based metrics may be a more robust parameter for assessing vascular reactivity because of reflecting overall velocity changes during the cardiac cycles rather than isolated velocity peaks (Khan et al., 2016; Leung et al., 2013; Liu et al., 2012). According to that, future studies using RT-PC MRI for CVRC-testing should also investigate CVRC based on mean flow velocities or mean flow volumes during different cardiac cycles in the standardized breathing protocol.

## 5 Conclusion

While RT-PC MRI effectively captured physiological blood flow profiles, it consistently underestimated flow velocities in intracranial arteries compared to nvUS, particularly in small and distal vessels. Despite this limitation, RT-PC MRI reliably detected dynamic changes in cerebral blood flow and proved valuable for assessing CVRC in various intracranial vessel locations. These findings position RT-PC MRI as a promising alternative for evaluation of CVRC. Future studies should investigate its application across diverse intracranial regions and in patients with intracranial artery stenosis.

## CRediT authorship contribution statement

**Sabine Hofer:** Supervision, Methodology, Formal analysis, Conceptualization, Software, Visualization, Writing – review & editing, Writing – original draft. **Peter Dechent:** Supervision, Methodology, Conceptualization, Data curation, Project administration, Software, Writing – review & editing, Writing – original draft. **Mathias Bähr:** Supervision, Conceptualization, Project administration, Resources. **Ilko Maier:** Writing – review & editing, Validation, Supervision, Project administration, Methodology, Investigation, Formal analysis, Data curation, Conceptualization, Writing – original draft.

## Funding

We acknowledge support by the Open Access Publication Funds of the Göttingen University.

## Declaration of competing interest

The authors declare that they have no known competing financial interests or personal relationships that could have appeared to influence the work reported in this paper.

## Figures and Tables

**Figure:**
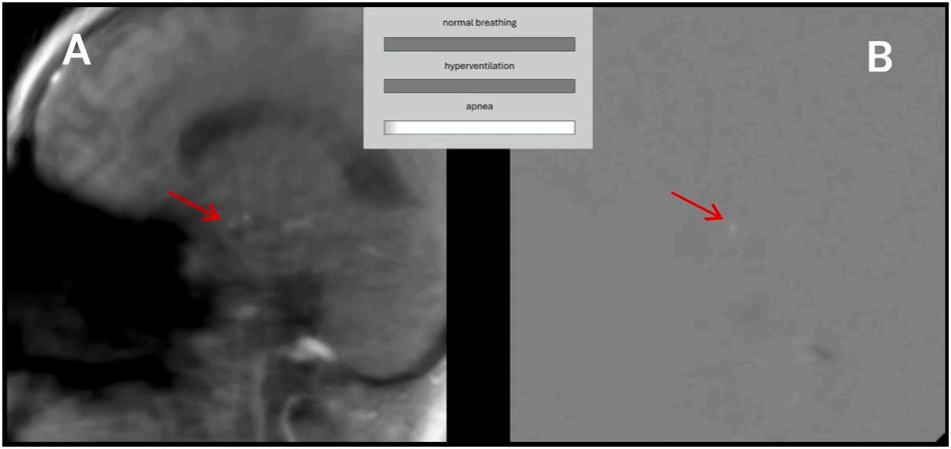


## Data Availability

Data will be made available on request.

## Video Files

Video 1: CVRC-testing of the MCA with RT-PC MRI.

Magnitude- (A) and phase-contrast-maps (B) of RT-PC MRI derived CVRC-testing in the MCA. The red arrows mark the MRI-signal of the MCA. In the middle of the video breathing instructions are depicted as the subjects saw them in a similar presentation during the MRI investigation. During hyperventilation the MRI signal of the MCA shows a less hyperintense signal due to vasoconstriction and decreasing carbon dioxide levels. During the end of apnea, the MRI signal of the MCA represents a strong hyperintense signal due to vasodilatation and increased carbon dioxide levels.

Video-Still (Thumbnail):
