# Supplementary material for: Real-time phase-contrast MRI can be used to quantify cerebrovascular reserve capacity – a comparative study to neurovascular ultrasound in healthy subjects
Source: Neuroimage Rep. 2026 Feb 25;6(1):100332. doi: 10.1016/j.ynirp.2026.100332 (PMC13080656; doi:10.1016/j.ynirp.2026.100332)
Supplement: Multimedia component 1 [file mmc1.docx]

**Supplementary material for “Real-Time Phase-Contrast MRI can be used to quantify cerebrovascular reserve capacity – a comparative study to neurovascular ultrasound in healthy subjects”**

**Supplementary Table 1:** Paired t-test of EDV and RI in the intracranial vessels with the RT-PC MRI and nvUS.

| vessel | nvUS  (mean cm/s ± standard deviation) | RT-PC MRI  (mean cm/s ± standard deviation) | difference  (mean cm/s ± standard deviation) | p-value | 95%-confidence-interval |
| --- | --- | --- | --- | --- | --- |
| **EDV** | | | | | |
| MCA-L | 54 ± 9.5 | 18.5 ± 5.5 | 35.6 ± 10.7 | <0.001 | 30.8, 40.3 |
| MCA-R | 54 ± 13.7 | 14.8 ± 4.9 | 39.2 ± 13.6 | <0.001 | 32.8, 45.6 |
| ACA-L | 28.8 ± 10.2 | 11.3 ± 2.8 | 17.5 ± 9.9 | <0.001 | 12.8, 22.3 |
| ACA-R | 22.4 ± 10.4 | 11.4 ± 3.7 | 10.8 ± 11 | <0.001 | 5.8, 16.1 |
| PCA-L | 44.8 ± 16.7 | 8.5 ± 2.3 | 36.3 ± 15.7 | <0.001 | 29.6, 42.9 |
| PCA-R | 41.5 ± 13.1 | 8 ± 2.3 | 33.5 ± 13.1 | <0.001 | 28, 39 |
| BA | 28.9 ± 9.6 | 18.7 ± 4.9 | 10.2 ± 8.6 | <0.001 | 6.7, 13.8 |
| VA-L | 24.1 ± 7.5 | 11.4 ± 4.1 | 12.7 ± 7.1 | <0.001 | 9.6, 15.8 |
| VA-R | 26.2 ± 8.9 | 12.2 ± 5 | 14 ± 9.5 | <0.001 | 10.1, 17.9 |
| ICA-L | 33.5 ± 9.8 | 16.6 ± 5.2 | 16.9 ± 8.2 | <0.001 | 13.5, 20.3 |
| ICA-R | 30.6 ± 7 | 16.8 ± 5 | 13.8 ± 7.3 | <0.001 | 10.8, 16.8 |
| **RI** | | | | | |
| MCA-L | 0.6 ± 0.1 | 0.3 ± 0.1 | 0.2 ± 0.1 | <0.001 | 0.2, 0.3 |
| MCA-R | 0.6 ± 0.1 | 0.3 ± 0.1 | 0.2 ± 0.1 | <0.001 | 0.2, 0.3 |
| ACA-L | 0.6 ± 0.1 | 0.3 ± 0.1 | 0.3 ± 0.1 | <0.001 | 0.2, 0.3 |
| ACA-R | 0.6 ± 0.1 | 0.4 ± 0.1 | 0.3 ± 0.1 | <0.001 | 0.2, 0.3 |
| PCA-L | 0.6 ± 0.1 | 0.4 ± 0.1 | 0.1 ± 0.1 | <0.001 | 0.1, 0.2 |
| PCA-R | 0.6 ± 0.1 | 0.4 ± 0.1 | 0.1 ± 0.1 | <0.001 | 0.1, 0.2 |
| BA | 0.6 ± 0.1 | 0.5 ± 0.1 | 0.1 ± 0.2 | <0.001 | 0.1, 0.2 |
| VA-L | 0.6 ± 0 | 0.5 ± 0.1 | 0.1 ± 0.1 | <0.001 | 0.1, 0.2 |
| VA-R | 0.5 ± 0.1 | 0.5 ± 0.1 | 0.1 ± 0.1 | 0.003 | 0, 0.1 |
| ICA-L | 0.6 ± 0.1 | 0.4 ± 0.1 | 0.2 ± 0.1 | <0.001 | 0.1, 0.2 |
| ICA-R | 0.6 ± 0.1 | 0.4 ± 0.1 | 0.2 ± 0.1 | <0.001 | 0.1, 0.2 |

Values are described as mean ± standard deviation. Vessel locations are given as left (L) or right site (R). RT-PC MRI: real-time phase-contrast magnetic resonance imaging; nvUS: neurovascular ultrasound; EDV: end-diastolic velocity; RI: resistance index; MCA: medial cerebral artery; ACA: anterior cerebral artery; PCA: posterior cerebral artery; BA: basilar artery; VA: vertebral artery; ICA: intracranial carotid artery.

**Supplementary Table 2**: Correlation analysis between nvUS and RT-PC MRI for flow velocity measurements of the intracranial arteries

| vessel | correlation-coefficient | p-value |
| --- | --- | --- |
| **PSV** | | |
| MCA-L | -0.109 | 0.63 |
| MCA-R | 0.098 | 0.68 |
| ACA-L | 0.286 | 0.235 |
| ACA-R | -0.019 | 0.936 |
| PCA-L | 0.238 | 0.263 |
| PCA-R | 0.105 | 0.627 |
| BA | 0.074 | 0.726 |
| VA-L | 0.335 | 0.118 |
| VA-R | 0.194 | 0.354 |
| ICA-L | 0.414 | 0.039 |
| ICA-R | -0.006 | 0.977 |
| **EDV** | | |
| MCA-L | 0.054 | 0.811 |
| MCA-R | 0.197 | 0.404 |
| ACA-L | 0.239 | 0.324 |
| ACA-R | 0.003 | 0.99 |
| PCA-L | 0.498 | 0.013 |
| PCA-R | 0.11 | 0.61 |
| BA | 0.445 | 0.026 |
| VA-L | 0.367 | 0.085 |
| VA-R | 0.151 | 0.471 |
| ICA-L | 0.546 | 0.005 |
| ICA-R | 0.3 | 0.145 |
| **RI** | | |
| MCA-L | 0.017 | 0.94 |
| MCA-R | 0.067 | 0.778 |
| ACA-L | 0.167 | 0.495 |
| ACA-R | -0.488 | 0.029 |
| PCA-L | 0.445 | 0.029 |
| PCA-R | -0.172 | 0.423 |
| BA | -0.285 | 0.167 |
| VA-L | -0.191 | 0.382 |
| VA-R | -0.14 | 0.503 |
| ICA-L | 0.027 | 0.896 |
| ICA-R | -0.304 | 0.14 |

Values are averaged across subjects. Vessel locations are given as left (L) or right site (R). RT-PC MRI: real-time phase-contrast magnetic resonance imaging; nvUS: neurovascular ultrasound; PSV: peak-systolic velocity; EDV: end-diastolic velocity; RI: resistance index; MCA: medial cerebral artery; ACA: anterior cerebral artery; PCA: posterior cerebral artery; BA: basilar artery; VA: vertebral artery; ICA: intracranial carotid artery.

**Supplementary Table 3:** ICC for the measurement of PSV in intracranial arteries derived by nvUS and RT-PC MRI.

| parameter | ICC | p-value | 95%-KI |
| --- | --- | --- | --- |
| RT-PC MRI | 0.744 | <0.001 | 0.546, 0.856 |
| nvUS | 0.886 | <0.001 | 0.805, 0.933 |

Values are averaged across subjects. ICC: intraclass-correlation; PSV: peak-systolic velocity; nvUS: neurovascular ultrasound; RT-PC MRI: real-time phase-contrast magnetic resonance imaging.

**Supplementary Table 4:** Paired t-test of arterial inflow and venous outflow volumes in ml/min derived by RT-PC MRI

|  | arterial inflow (ICA + VA) | venous outflow (transversal sinus) | difference | p-value | 95%-KI |
| --- | --- | --- | --- | --- | --- |
| t-test | 434.4 ± 156.2 | 465.6 ± 121 | 31.2 ± 205 | 0.454 | -53.4, 115.8 |

Values are averaged across subjects. ICA: intracranial carotid artery; VA: vertebral artery; RT-PC MRI: real-time phase-contrast magnetic resonance imaging.

**Supplementary Table 5:** Descriptive comparison of flow measurements in venous sinus to literature (Mehta et al., Stoquart-Elsankari et al.)

| vessel | RT-PC MRI | 2D-PC MRI (Mehta et al., 2000; Stoquart-Elsankari et al., 2009) |
| --- | --- | --- |
| **velocity max. (cm/s)** | | |
| SSS | 31.4 ± 6.4 | 15.3 ± 3.5 |
| TS-R | 20.4 ± 5.6 | 13 ± 5.8 |
| TS-L | 19.5 ± 6.5 | 10.8 ± 4.3 |
| **flow volume (ml/min)** | | |
| SSS | 379.6 ± 69.5 | 335 ± 57 |
| TS-R | 291.3 ± 126.2 | 434 ± 204 |
| TS-L | 221.4 ± 100.9 | 195 ± 152 |

Values are given as mean ± standard deviation. Vessel locations are given as left (L) or right (R) site. RT-PC MRI: real-time phase-contrast magnetic resonance imaging; 2D-PC MRI: 2-dimensional phase-contrast magnetic resonance imaging; SSS: superior sagittal sinus; TS: transversal sinus.

**References**

Mehta, N.R., Jones, L., Kraut, M.A., Melhem, E.R., 2000. Physiologic Variations in Dural Venous Sinus Flow on Phase-Contrast MR Imaging. AJR Am J Roentgenol 175, 221–225. https://doi.org/10.2214/ajr.175.1.1750221

Stoquart-Elsankari, S., Lehmann, P., Villette, A., Czosnyka, M., Meyer, M.-E., Deramond, H., Balédent, O., 2009. A Phase-Contrast MRI Study of Physiologic Cerebral Venous Flow. J Cereb Blood Flow Metab 29, 1208–1215. https://doi.org/10.1038/jcbfm.2009.29
